# Preoperative evaluation using external lumbar drainage for patients with posthemorrhagic hydrocephalus: A prospective, monocentric, randomized controlled trial

**DOI:** 10.1097/MD.0000000000021872

**Published:** 2020-08-28

**Authors:** Tong Sun, Junwen Guan, Jingguo Yang, Yikai Yuan, Yicheng Zhou, Chao You

**Affiliations:** a Department of Neurosurgery; b West China Brain Research Center; c Neurosurgery Research Laboratory, West China Hospital, Sichuan University, Chengdu, Sichuan, P.R China.

**Keywords:** clinical outcomes, comparison, external lumbar drainage, lumboperitoneal shunt, post-hemorrhagic hydrocephalus, randomized controlled trial

## Abstract

**Background:**

External lumbar drainage (ELD) remains the most common used methods with a higher sensitivity before lumboperitoneal shunt (LPS) implantation to predict the shunt outcomes in the treatment of idiopathic normal pressure hydrocephalus. However, the benefits of such supplemental test have not been tested in the treatment of post-hemorrhagic hydrocephalus (PHH).

**Methods and design:**

In the current trial, 100 eligible patients with PHH will be recruited and randomly assigned to the ELD group (study group) and non-ELD group (control group). Lumbar puncture (LP) will be performed for participants in non-ELD group. LP plus ELD will be performed for participants in ELD group, those who will then be investigated the suitability of potential LPS 4 days after ELD. Two independent and practiced assessors will collect the baseline data and evaluate each participant 4 days after ELD or LP, 1 day after LPS, at the time of discharge and 1 month after LPS. The primary outcome is the shunting outcomes 1 month after surgery. The secondary outcomes include the complications related to ELD, complications related to LPS, and Evens index at each evaluation point. Meanwhile, serious adverse events occurring at any time is recorded to determine the safety of this trial.

**Discussion:**

The results of this trial will demonstrate whether preoperative evaluation using temporary ELD for patients with PHH could attenuate the risk of LPS failure.

**Trial registration number:**

ChiCTR2000034094; Pre-results.

## 1 Introduction

Since the application of shunt surgery in clinic, a great deal of attention was directly given to quest the path to attenuate the incidence of shunt failure.^[1,2]^ To date, preoperative accurate evaluation through supplemental test has been widely accepted to correctly select the suitable patients for shunt implantation.^[3,4]^ In this regard, external lumbar drainage (ELD) remains the most common used methods with a higher sensitivity before lumboperitoneal shunt (LPS) implantation to predict the shunt outcomes in the treatment of idiopathic normal pressure hydrocephalus.^[5]^ However, the benefits of such supplemental test have not been tested in the treatment of post-hemorrhagic hydrocephalus (PHH), a common disease occurring secondary to intracranial hemorrhage.^[6]^

## 2 Methods and design

### 2.1 Objective

To prove whether preoperative evaluation using ELD for patients with PHH could attenuate the risk of LPS failure.

### 2.2 Study design

**Figure 1:**
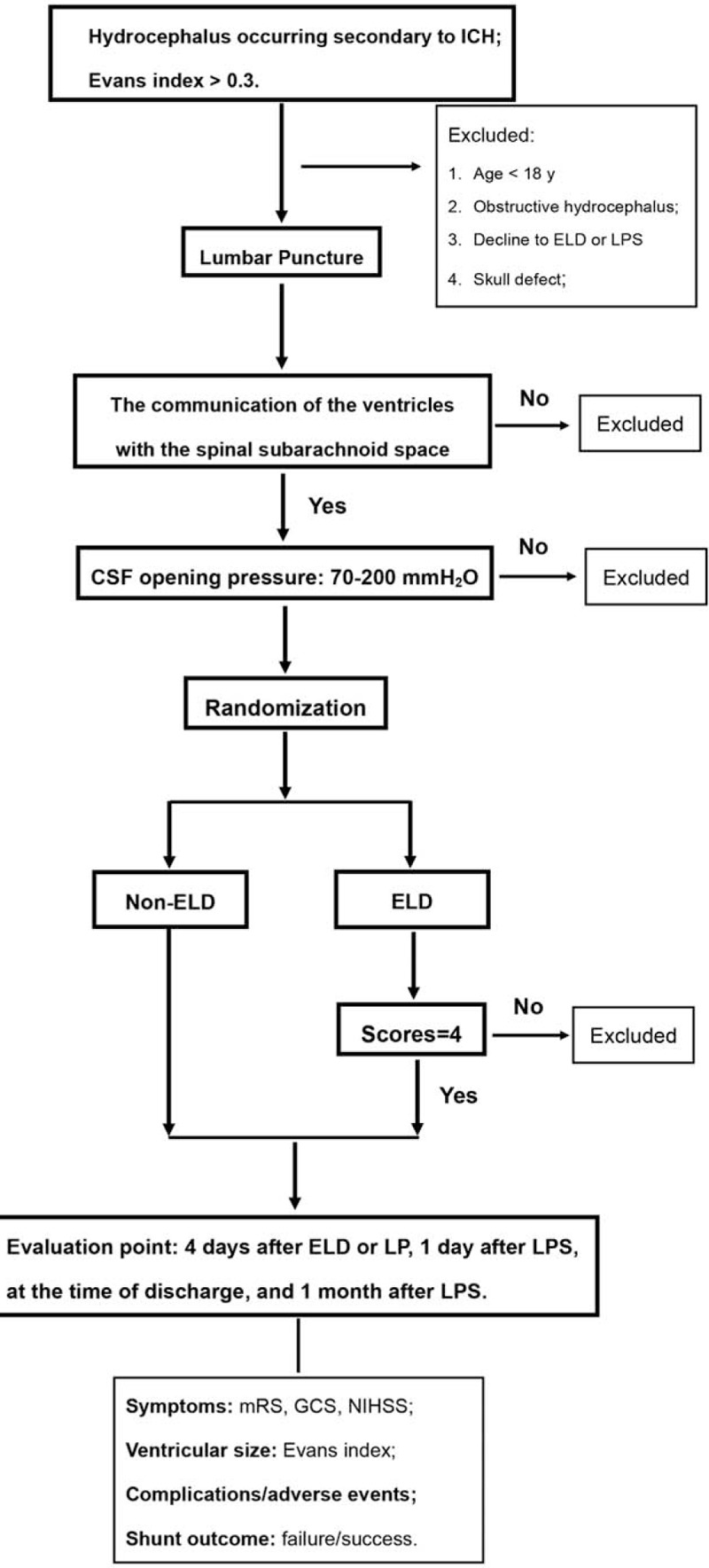
Flow chart of the selection pf patients. CSF = cerebrospinal fluid, ELD = external lumbar drainage, GCS = Glasgow coma scale, ICH = intracranial hemorrhage, LPS = lumboperitoneal shunt, mRS = modified Rankin Scale, NIHSS = National Institute of Health stroke scale.

The flow chart of the selection of patients is shown in Figure 1. In the current trial, 100 eligible patients with PHH will be recruited from the Department of outpatient of Sichuan University West China Hospital since September 1, 2020, and randomly assigned to the ELD group (study group) and non-ELD group (control group). Lumbar puncture (LP) will be performed for participants in non-ELD group. LP plus ELD will be performed for participants in ELD group, those who will then be investigated the suitability of potential LPS 4 days after ELD. Two independent and practiced assessors will collect the baseline data and evaluate each participant 4 days after ELD or LP, 1 day after LPS, at the time of discharge and 1 month after LPS. The primary outcome is the shunting outcomes 1 month after surgery. The secondary outcomes include the complications related to ELD, complications related to LPS, and Evens index at each evaluation point. Meanwhile, serious adverse events occurring at any time is recorded to determine the safety of this trial.

### 2.3 Recruitment and eligibility

#### 2.3.1 Inclusion criteria

1. Age >18 years;
2. Ventricular expansion occurring secondary to intracranial hemorrhage;
3. Evans index >0.3;
4. LP indicates that the subarachnoid space of spinal cord is connected with ventricles, and the cerebrospinal fluid opening pressure is between 70 and 200 mm Hg.

#### 2.3.2 Exclusion criteria

1. Obstructive hydrocephalus;
2. Decline to ELD or LPS surgery;
3. Skull defect.

### 2.4 Sample size

Previously published reports indicated that the rate of LPS failure for patients with preoperative evaluation using ELD was 15.7%, comparing with a rate of 40.9% for patients without ELD.^[7]^ In this regard, a sample of 45 will be required in this trial with a significance level of 5% (2-sided) and a power of 80% to demonstrate a 20% difference in rate of shunt failure. Considering about the loss to follow-up, the sample size is enlarged to 50 for each group.

### 2.5 Randomizing and blinding

The randomization will be performed using a random number table that is generated by the statistical program SAS Version 9.4 (SAS Institute Inc, Cary, NC) and is kept secret by the statisticians who are independent of this study. This trial is open-label, but assessors and analysts are blind to allocation and the intervention clinicians will not involve in any assessment.

### 2.6 Intervention

Physicians, neurosurgeons, and clinicians will be trained centrally in advance and reach uniform standard.

### 2.7 Temporary ELD

ELD is performed under local infiltration anesthesia and the patients are positioned in the left lateral position. A lumbar catheter is inserted through the L3/4 or L2/3 interlaminar space into the spinal subarachnoid space and then connected to the drainage system. According to previous studies, the drainage period is 3 days and drainage volume is 150 to 200 mL per 24 hours.^[8]^

### 2.8 Preoperative evaluation

Repeated evaluation will be performed using the scale, as shown in Table 1, 4 days after ELD to investigate the suitability of potential LPS and then the ELD system will be removed. Patients with scores 4 will be included in this trial and the remaining patients will be excluded. Specifically, the improvement of clinical manifestation is defined as an improvement of 1 point or more in the National Institute of Health stroke scale, Glasgow coma scale, modified Rankin scale, or according to self-assessment.

**Table 1:**
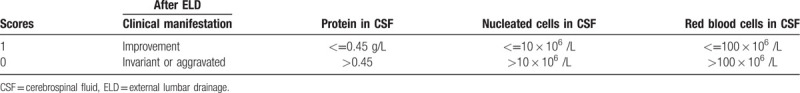
Preoperative evaluation scale after ELD.

### 2.9 Outcomes

Two independent and practiced assessors will collect the baseline characteristics and evaluate each participant 4 days after ELD or LP, 1 day after LPS, at the time of discharge and 1 month after LPS. The evaluation schedule is shown in Table 2.

**Table 2:**
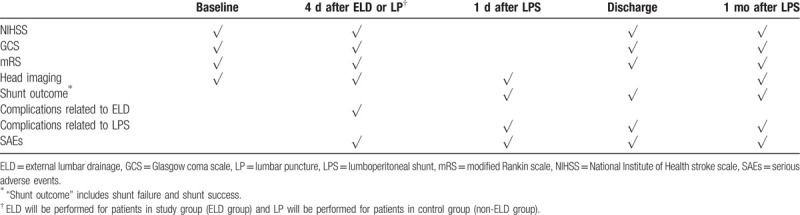
Study schedule.

#### 2.9.1 Primary outcome

The primary outcome is the shunting outcomes 1 month after surgery. Shunting outcomes include shunt failure and shunt success. According to previous studies, which is defined as the shunt obstruction, breakage, tubing exposure, malfunction, or infection requiring shunt revision. Shunt success is defined as the absence of shunt failure.

#### 2.9.2 Secondary outcomes

The secondary outcomes include the complications related to ELD, complications related to LPS, and Evens index at each evaluation point.

Meanwhile, considering about the safety of this trial, serious adverse events occurring at any time is recorded.

### 2.10 Data collection

Two independent and practiced assessors will collect the data. All data will be recorded in the paper-based Case Record Form and Electronic Data Capture. A third reviewer is required while there are any debates on data collection.

### 2.11 Data and safety monitoring

Members of independent data monitoring committee, including 1 physician, 1 statistician, and 1 data analyst, are responsible for monitoring the safety and efficacy of this trial once a month.

### 2.12 Statistics analysis

SPSS version 19 (IBM, Armonk, NY) is used to analyze statistics and a *P*-value (2-sided) under .05 is considered to have statistical difference. Kolmogorov–Smirnov test is first used to determine the normality of quantitative data. Quantitative data followed normal-distribution are statistically described as arithmetic mean ± standard deviation. Other quantitative data (non-normal distribution) are statistically described as median (range). Categorical data, such as sex, shunting outcomes, and complications, are statistically descried as number (percent). To compare the 2 groups on quantitative data followed normal-distribution, independent samples *t-*test is used. To compare the 2 groups on the other quantitative data, Wilcoxon rank sum test is used. To compare the 2 groups on categorical data followed normal-distribution, Chi-square test is used.

### 2.13 Withdrawal and dropout

If at least 2 researchers judge that it is not appropriate to continuously participant in this trial, or any participants choose withdrawal and dropout, the reasons of withdrawal and dropout will be recorded and submitted to the supervisor for review.

## 3 Discussion

This study will be the first randomized controlled trial that analyzes the clinical outcomes of patients diagnosed as PHH with or without evaluation using temporary ELD before LPS implantation. The results of this trial will demonstrate whether preoperative evaluation using temporary ELD for patients with PHH could attenuate the risk of LPS failure and generate the discussion about the accurate evaluation of the suitability of shunt surgery through supplemental test. Additionally, the trial will provide the evidence for the association of shunt failure with the cerebrospinal fluid count and protein level.

The current trial, however, still has some limitations. First, it is a single-center study. Second, medical conditions and surgeons’ experiences are contributed to the postoperative outcomes. In this regard, personnel will be trained centrally in advance and reach uniform standard.

## Author contributions

**Conceptualization:** Junwen Guan, Chao You, Tong Sun.

**Data curation:** Yikai Yuan, Jingguo Yang.

**Funding acquisition:** Chao You.

**Investigation:** Tong Sun, Junwen Guan, Yicheng Zhou.

**Statistical analysis:** Yikai Yuan, Jingguo Yang.

**Study design:** Tong Sun, Junwen Guan.

**Supervision:** Chao You.

**Validation:** Chao You.

**Writing – original draft:** Tong Sun.

**Writing – review & editing:** Junwen Guan, Chao You, Yicheng Zhou.
